# Perceptions of Factors Associated With Sustainability of Health Care Innovation Centers

**DOI:** 10.1001/jamanetworkopen.2023.39129

**Published:** 2023-10-27

**Authors:** Holly Krelle, Michael Martinez, Kira Garry, Leora I. Horwitz

**Affiliations:** 1 Center for Healthcare Innovation and Delivery Science, NYU Langone Health, New York, New York; 2 Department of Population Health, NYU Grossman School of Medicine, New York, New York; 3 Department of Medicine, NYU Grossman School of Medicine, New York, New York; 4 Penn State College of Medicine, State College, Pennsylvania

## Abstract

**Importance:**

Innovation centers in US health care systems are increasingly common but variably successful and sustainable. Few studies have examined how and why some centers achieve sustainable success but others do not.

**Objective:**

To explore whether and how innovation centers in US health care systems are able to successfully sustain themselves over multiple years.

**Design, Setting, and Participants:**

In this qualitative study, semistructured, qualitative interviews of leaders at 9 innovation centers across the US were conducted from January 1, 2019, to December 31, 2020. Data analysis was conducted from December 2020 to April 2021 using qualitative methods and a deductive framework based on the Consolidated Framework for Implementation Research. Centers were identified through purposeful sampling. The 9 study centers had a mean age of 6 years (range, 2-15 years); most were affiliated with an academic teaching hospital and half with a medical school. Two were wholly separate from the health care system. Two-thirds had fewer than 10 full-time staff members.

**Exposures:**

All interviewees were staff of a US-based innovation center. This study did not evaluate particular interventions or innovations.

**Main Outcomes and Measures:**

Perceptions of and views on factors associated with the success and sustainability of innovation centers.

**Results:**

Staff interviewed at 9 innovation centers across the US described 3 key activities that appeared to be associated with long-term sustainability: facilitating innovation projects that were valued for quality improvement and cost avoidance, not just return on investment; acting as networking nodes for their institutions; and upskilling staff. These activities were associated with improved institutional culture. Two structural characteristics underpinned successful centers: finding an effective balance between being “internal” and being “external” to the organization and providing practical support and skills otherwise lacking within the wider institution.

**Conclusions and Relevance:**

This qualitative study of 9 innovation centers explored how centers sustained themselves within US health care institutions and showed that success was often associated with interpersonal relationships and cultural benefits. Independent financial sustainability was not always essential to longevity; systems also valued how centers could create cultures of innovation and upskill staff.

## Key Points

**Question:**

What makes a health care innovation center sustainable?

**Findings:**

The centers’ employees interviewed in this qualitative study described 4 key factors associated with sustainability: facilitating innovation projects; acting as networking nodes for their institutions; upskilling staff; and creating a culture that is supportive of innovation. Two characteristics appeared to underpin sustainability: finding an effective balance between being “internal” and being “external” to the organization and providing practical support and skills otherwise lacking within the wider institution.

**Meaning:**

This study suggests that innovation centers can sustain themselves within US health care institutions, but centers in this study found their success to be associated more with interpersonal relationships and cultural benefits than with financial returns.

## Teaser

This qualitative study assesses the perceptions of employees of innovation centers within US health care systems on whether and how such centers are able to successfully sustain themselves over multiple years.

## Introduction

Innovation centers are becoming increasingly common in US health care systems. Through a variety of methods (design thinking, rapid randomized trials, and agile software development) and partnerships, most innovation centers aim to quickly develop, operationalize, and robustly evaluate new innovations (often digital) within a health care system.^1^ Previous work has defined innovation centers as collaborative spaces that bring together heterogenous expertise.^2^

Innovation centers are typically built in response to health care systems’ struggles.^3^ Centers may have diverse aims but commonly focus on real-time health care delivery and problem solving^4^ by embracing 3 core activities: (1) using data, experiences, and activities to generate new solutions and ideas; (2) spreading innovative, successful ideas from one part of the health care system to another; and (3) rapidly testing, evaluating, and scaling homegrown interventions.

When functioning well, innovation centers can offer high-quality, hyperlocal research and quality improvement.^5^ Effective centers are structured to allow for the robust analytical and evaluative support often found in academic settings to be combined with the highly pragmatic and operations-focused ethos of the hospital center.^6^ However, it is estimated that up to 90% of industry innovation centers fail.^7^

Health care innovation centers have experienced unprecedented growth in the past 10 years. A 2018 Becker’s Hospital Review survey identified 66 hospitals and health systems with active innovation programs.^8^ More recent estimates suggest that the number is now nearer 110.^9^ However, evaluation to date has remained relatively light, with minimal exploration of how centers define success and achieve sustainability. This study explores the characteristics of 9 successful US innovation centers, focusing on how these centers defined and achieved success and sustainability.

## Methods

### Study Design

We conducted an exploratory qualitative study of innovation centers in the US using semistructured qualitative interviews conducted from January 1, 2019, to December 31, 2020. The interviews focused on understanding how the centers implemented their innovations, exploring what worked and what did not. We obtained approval for this study from the institutional review board at NYU Langone Health. Each participant provided verbal informed consent. This study followed the Standards for Reporting Qualitative Research (SRQR) reporting guideline.

### Sampling

Centers were identified via 3 sources: a 2015 survey of innovation centers by the Commonwealth Fund,^1^ an internet search, and snowball sampling from interviewees. The study is qualitative and exploratory. Our sampling approach therefore aimed for saturation rather than representativeness.^10^ We began with an initial long list of centers, contacting subsequent centers as participants agreed or declined to participate. We purposively sampled for maximum variation based on differences in centers’ age, structure, funding sources, aims, and size. Interviewing continued until we reached saturation (limited new information was uncovered), in line with qualitative methods.

### Data Collection

Data collection for each center consisted of semistructured, in-depth interviews. Interviews were with either the directors of each center or a designated representative, with some interviews including an additional person in a leadership role. Each interviewee was interviewed once initially; some were interviewed a second time if further clarification was needed. All interviews were completed by telephone or video call.

In line with SRQR guidelines,^10^ interviews were conducted using a topic guide (eAppendix 1 in Supplement 1) exploring themes around centers’ structure and funding, aims and objectives, key ingredients of implementation success, and achievements. All interviews were led by the corresponding author (H.K.), sometimes accompanied by another author (L.I.H). Interviews were, with permission, digitally audio-recorded and transcribed using Otter AI software, version 2020 (Otter.ai). The data collection tools did not change during the study. Data generation ended when saturation was reached and when no significant new themes emerged.

### Statistical Analysis

Data analysis was conducted from December 2020 to April 2021. Qualitative data analysis was conducted when interviewing was complete. We took a deductive approach, basing our initial code structure on the Consolidated Framework for Implementation Research,^11^ a well-established framework used extensively in implementation research. The framework provides a menu of constructs across 5 domains: the inner setting, the outer setting, the individuals, the process, and the intervention itself. This framework provided an overall structure, but during coding, we allowed new themes to emerge and refined the framework accordingly. Some themes were rarely spoken about or covered elsewhere and are therefore excluded from the Results section. The full framework is shown in eAppendix 2 in Supplement 1.

Interview transcripts were hand coded and matched against the framework using Excel 2016 (Microsoft Corp). Coding was conducted by 3 of us (H.K., M.M., and K.G). All researchers independently coded the same 2 interviews, which were then discussed and compared to find divergent views as well as to standardize the coding process. Researchers then coded their remaining transcripts. Detailed within-center analysis was followed by cross-center analysis to identify overarching themes, similarities, differences, and transferable lessons learned.

## Results

### Overview of the Centers

Eleven employees from 9 health innovation centers across the United States were interviewed about their inner settings, outer settings, processes, failures, and challenges (eAppendix 3 in Supplement 1). The mean number of years that the centers interviewed have been in existence was 6 (range, 2-15). Most centers were affiliated with an academic teaching hospital, and 3 were also attached to a medical school (Figure). Three were associated with public hospitals, and 2 were outside the governance of the medical center. Centers were generally quite small; 3 centers had fewer than 10 people.

**Figure.**
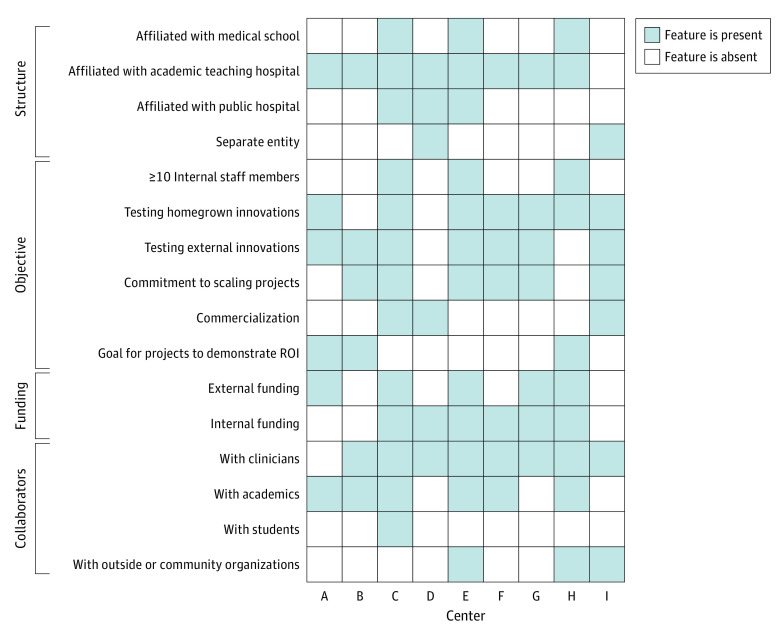
Overview of the Centers ROI indicates return on investment.

### Defining and Achieving Sustainability

Herein, we highlight 3 activities or functions of centers that were commonly reported as being key to sustainability, as well as 2 structural characteristics that underpinned centers’ ability to carry out these activities. The Table summarizes key quotes supporting each of the identified themes. Our deductive approach to analysis meant that these themes are not a systematic representation of success but rather an emerging, common set of themes identified by interviewees as most important in their contexts. We found that centers commonly saw themselves as having 3 functions: (1) facilitating individual projects, which were often evaluated not by return on investment but by other aspects of value (such as quality improvement or cost avoidance); (2) acting as networking nodes for their institutions; and (3) upskilling staff. The centers’ interviewees described these activities as being collectively associated with institutional culture change, an overarching benefit that was key to sustainability. Two structural characteristics underpinned successful centers, from the perspective of center directors: a balance between being “internal” and being “external” to the organization and an ability to provide practical support and skills otherwise lacking within the wider institution.

**Table.** Key Quotes

Overarching themes and subthemes	Representative quotes
Intervention characteristics	“We were really involved at the ground level in the planning process. The health system wanted to do something about hypertension…. And we met with the medical director, figured out what was feasible: we mapped it out together. So there really was buy-in and ownership on both sides.”
Beyond the hospital	“So keeping that entrepreneurial mindset, everything we do is really important, because I’m a big believer that you cannot have true impact without commercialization.… So if you can’t give it to the patient, then they can’t buy it. So there’s just no, there’s no cycle, or any ability to scale because the way our system is set up. So you know, we have ideas that are like, hey, my IV pole keeps breaking at this one point. And [company name] is saying that they’re not making these IV poles anymore. And if we want to fix them, then we have to buy IV pumps that come with those poles. So a 10-cent fix ends up being a $10 000 fix. So we know we have people come in and say hey, can you can you 3-D print this hook for us? Which, you know, in the in the grand scheme, innovation enthusiasts would say no, that’s not innovation. And I say, Well, have you ever worked at the bedside before? And have you ever had to, you know, duct tape something together to save someone’s life, because I would argue that that ends up being innovation.”
Outer setting (structures and networks; culture; organizational incentives, objectives, or priority put on innovation; financing; ROI; leadership engagement; and other)	Structures and networks: “So we wanted to build this institute more like a tech company would run. And to have incredible flexibility on work and how we work in teams, and approach types of people we bring in. That was very different from the hospital. And so being separate allowed us to be very flexible. We didn’t use any of the work policies and procedures of our system, necessarily. Now the second part, though, was we still wanted access to the hospital and [to] be considered part of that family and ecosystem. So that we could move our work quickly through the health system in partnership with clinicians and others there. And so we were able to get the right balance of interdependence.” “We need to have nurses talk to dancing majors, and we need to have physicians talk to archaeologists.” Organizational incentives: “We’re really trying to align our efforts with strategic priorities across the hospitals, so I guess I would say the categories that we set up when we do that proposal process have become a little bit more tailored each year to the strategic goals of the of the system. So I think that also helps kind of when we’re presenting in front of the senior leadership team that we were talking about, usually presents in buckets that are like, okay, these are the ones related to whatever priority. And that sort of also reinforces you know, that we’re not just doing this, like, oh, this sounds like a cool idea that we want to do, like, we have a reason [that] is aligned with this goal. And then we tried to do the patient metrics and the financial metrics to back it up.”
Inner setting (team structure, culture and ways of working, location within or outside hospital structure, access to knowledge and information or data, objectives, or other) in the wider hospital	Team structure: “We are about 16, faculty and staff, so it’s 10 faculty, 6 staff members…. So it probably comes out to about 5 or 6 FTE faculty, even though there’s more by number. And, you know, we also have an affiliate network. So there’s about another 15 or 20 affiliates. They don’t get specific grant support, they get pilot funds and mentorship.” Culture and ways of working: “I think the way that we approach things is with a mixed methods perspective on almost every project. So, designing some quantitative measure of change, putting implementation science outcomes on each project. So you know, how, what is your outcome really, that you’re looking at? Adoption? Or is it, you know, effectiveness, and everyone is always mixing those up? …I think we need still more qualitative analyst bandwidth. There’s, there’s enough quantitative people around to do most of the quantitative work. But most of the trouble we have is with qualitative work, the other types of people, I would like to add our people that have more econometrics type, quantitative experience; our traditional statisticians within the school of medicine or school of public health are actually not that familiar with many of the methods that we use for our designs.” Objectives: “So our innovation center is focused on innovation, it’s not focused on commercialization. And we have built a structure that allows innovation to focus at the micro level, and then potentially has the ability to flourish beyond that. But that’s not the goal. The goal is to create a structure of innovation that allows a culture of innovation to thrive.”
Characteristics of individuals involved in the center itself	“And so that doesn’t include physicians, but that also includes nurses, respiratory therapists, psychologists, pharmacists, etc… it doesn’t have to be physicians, it can be any clinician, any administrator, any combination of a team. So anyone’s welcome to apply.” “And we’re able to recruit different types of people than you would normally do for hospital like software engineers. And folks like that, that continued about 3 or 4 years, and I was an indication of how it was structured, I was named the president and CEO of that research center, that institute. And it has separate board of directors, several independent board directors that we recruited from the outside.”
Process (planning, engaging, selecting, implementing, and evaluating)	Planning, engaging, and selecting: “We have a quick application process where we’re really trying to focus on the details of the intervention, the proposed intervention, and what you are going to track to see if it’s working, we really don’t want to encourage you know, the length of like a research paper, it’s supposed to be really short. So then we receive proposals over the summer, August has a pretty busy time where all the proposals are coming in, we’re reviewing them as a project team…. We have roughly about 10 projects per year, it kind of varies, you know, we have to align with strategic goals across the hospital and other things, but it’s on average about 10 a year. And then we pilot those projects for the rest of the fiscal year.” Evaluating: “Challenge over study design; tension between rigor and speed. Always a challenge to meet in the middle. But the rest of the team have data science or stats training. They have come to be tapped for program evaluation—other teams in the innovation are creating (eg, seeing they’re wanting to roll out a clinical disease remote monitoring platform)—precision health. Huge pressure to implement quickly across the board; but incompatible with evaluating it the way we want to.”
Examples of success and failure	Examples of success: “Right now, I’m running a public campaign with my nonprofit called [redacted]…. And, you know, that’s an innovation to us. That’s a policy innovation. That’s a social awareness. Innovation. But it’s not something that could ever be commercialized… still out there in the chaos, and we’re getting, we’re having collaborations and connections with people all over the country because of it. And that’s, that’s where we like to play.” Examples of failure: “Sometimes, like what I have, in particular in mind, what one of them was just, I think, bad timing… I think, you know, perhaps the, for this other particular one, we may have needed different, a different way to evaluate it…. Maybe in that case, we didn’t have the right evaluation metric, because it still had nuggets that we can learn from.”

Abbreviations: CEO, chief executive officer; FTE, full-time equivalent; IV, intravenous fluids; ROI, return on investment; 3-D, 3-dimensional.

#### Facilitating Projects and Identifying Their Nonmonetary Value

It is sometimes assumed that innovation centers must be financially self-sustaining or that a key aim of many centers is the commercialization of innovations. In reality, all center directors, even directors of centers that were more explicitly focused on commercialization, recognized that substantial financial returns were unlikely. Instead, centers focused on “demonstrating value in a crisp way” (center B) and on thinking more creatively about how their innovations and projects might add value for their institution. The interviewee of center H, for instance, described a project that “involved creating a decision support tool in the electronic health record, to identify patients who smoke when they got admitted to the hospital…. And I thought, I know it’s good for the patients, but that doesn’t really make much money for the institution. But what’s my hook going to be on this? So I was able to convince the institution that, if you automate this it will help you meet that quality metric… it will make you look good with, you know, Department of Health and Human Services, and the Joint Commission and CMS [Centers for Medicare & Medicaid Services] and all these other, you know, federal agencies and regulatory agencies that take a look at our tobacco treatment.”

For other centers, the value they offered was in “failing fast”—spending less money to understand whether a project or innovation would or would not work. This aspect often set them apart from the wider health care organization, which tended to be slower and more cautious. As the interviewee of center I discussed: “So that, you know, the failure isn’t a $10 million failure. Now, it might be a $10 000 failure… we talked to 100 consumers who said, you know what, I probably wouldn’t be willing to pay for something like this… [and] we just spent $3000 to figure that out. Versus back then it was the failure was, we put $10 million into innovation and nothing happened… we’re not making those, you know, big, multimillion dollar, quote unquote, failures. We’re gaining those failures much earlier in the process.”

#### Acting as Networking Nodes

Innovation often stems from connections. An often-unintended benefit of innovation centers is that they serve as a locus for individuals who otherwise would not have met to work together on new ideas. Interviewees reported that centers were a hub for that networking—they brought together people from disparate parts of the organization—and, in return, they helped create an overall organization that was better networked. As 2 interviewees noted:

“We like to say we’re the best dating service at [center G], because we help people get hooked up.… We sit firmly at the center of [center G], and over the years we’ve had the opportunity to interact with so many different key departments: we know a lot of people.” (Center G)

“We need to have nurses talk to dancing majors, and we need to have physicians talk to archaeologists.” (Center C)

This network could act as a virtuous circle, also benefitting the center itself. Several centers’ interviewees discussed how they used their network of “alumni” (people who had run projects with them in the past) as sources of both marketing and knowledge and to generate support for other projects.

#### Broadening Staff Skills and Outlooks

The recognition that commercialization was challenging meant that—for many centers—more immediate outcomes, such as upskilling and networking individual staff, became more important markers of success. As the interviewee from center F said, one of its key objectives was “to help clinicians in their efforts to change health care delivery, to help provide more robust methodological backbone, I think, to their efforts… [but also to] give a little bit more of a research background to their work, to help with learning, but also with dissemination, so to help them publish their findings, that would probably that’d be the first goal.”

### Culture Change

Ambitious centers did not just aim to change individual skills, they tried to shift the broader culture of their institution. Center directors reported that medical leadership often initially struggled with the pace and high failure rate of innovation centers. Business-focused leadership could be unwilling to engage with or properly understand evaluation methods or the nonmonetary value of quality improvement. Individual staff members might struggle with the practical skills or headspace to think of new ideas. By explicitly evaluating projects based on nonmonetary returns, connecting like-minded individuals across the institution, and creating human capital through training and repeated engagement in projects, successful centers could change institutional culture. Alumni of the program could often act as agents of change, spreading new ways of working and approaches to innovation throughout the institution. As the interviewee from center C put it, “our center is focused on innovation, it’s not focused on commercialization… the goal is to… allow a culture of innovation to thrive.”

Two structural features of center organization and practice were associated with centers’ ability to achieve success metrics. To act as a networking node and force for cultural change, successful centers had to balance being “internal” and “external” to their host organization.

Successful centers maintained a careful balance of being sufficiently embedded in the organization to have leverage and legitimacy, yet being separate enough to have flexibility, speed, and neutrality. There was often a tension between the robust, traditional ways in which hospitals and academia worked and the more innovative approaches of the centers themselves.

Being part of the wider hospital organization was a key advantage. As well as enabling them to act as a networking node, it gave centers legitimacy, clear ties to leadership, and real leverage. However, being part of the wider hospital organization could also be stultifying, limiting the creativity and speed with which people could move.

For others, being outside their parent organization was helpful—it gave them freedom from bureaucracy, the ability to move quickly, easier access to a more innovative way of working, and the ability to recruit staff who might not want to work at a more traditional academic or public institution. As the interviewee of one center put it: “so we wanted to build this institute more like a tech company would run. And to have incredible flexibility on work and how we work in teams and… the types of people we bring in. That was very different from a hospital.” (Center D)

However, even centers designed as an external group recognized the need to maintain strong connections with the parent hospital. Others noted the risk of being seen as outsiders who end up distant from crucial decisions. Balance and ongoing engagement were key.

“We still wanted access to the hospital and [to] be considered part of that family and ecosystem. So that we could move our work quickly through the health system in partnership with clinicians and others there. And so we were able to get the right balance of interdependence… so that we can kind of move more quickly… so there’s a little bit less constraints on promotions, hiring, firing.” (Center D)

“We do feel like outside consultants. At times we’re not in the boardroom when the key decisions are made. And you know, we have to align ourselves with what the health system priorities are without really [being] involved in the decision making.” (Center E)

Innovation was not just about encouraging people to have ideas—it was about providing practical support. Successful innovation centers did not just generate ideas or try to teach frontline staff to do everything themselves. Those that did well also saw themselves as wells of practical support, offering the kinds of technical support to would-be innovators (particularly clinical innovators) that they might otherwise lack. This worked to remove barriers that may have prevented the implementation of a practical project: “Kind of our core job is helping them operationalize their pilot… so how do they order supplies? Sort supply chain or vendor relations?” (Center B)

This practical support was also reflected in the staffing of many centers. Almost all interviewees emphasized not only the diversity of their staffing mix but also the inclusion of project management expertise “to get projects done” (center E). Some centers loaned these project managers to individual innovation projects, viewing that as a core part of their business offer. For others, this loaning centered around qualitative and quantitative evaluation expertise or engineering skills.

## Discussion

In this qualitative interview analysis, we found that innovation centers characterized their value broadly, beyond quantified financial return on investment or the creation of profitable innovations. Effective innovation centers were also recognized by their institutions as providing cultural benefit and building human and social capital as well as economic capital. They made innovation hyperlocal, avoided the well-established challenges and issues of scaling and transferring innovations from one location to another, and empowered individual hospital teams to fix their own problems while providing the technical support to help them do so.

Our findings echo wider work—both conceptual and practical—on what makes innovation centers successful in health care and across other industries. Our finding that effective innovation centers help to build organizational capacity and foster a culture of innovation is consistent with several other studies of what makes organizations successful at innovation more globally. For example, Weisberg et al^12^ frame innovation not as a specialist activity consigned to a special group of people in one part of an organization. Rather, innovation cultures are most successful when everyone is a potential innovator, supporting even “under-the-radar” innovators who simply need the skills and settings to support their idea. Cresswell et al^2^ found that successful centers worked hard to create organizational cultures that placed innovation at the core of their activities. Ahuja,^7^ discussing innovation laboratories across industries, concluded that the core of success is the extended innovation laboratory that views the upskilling of people as just as important as the development of innovations themselves.

We also found that innovations that improved institutional reputation or quality of care were of substantial value, even if not quantifiable through direct financial gain. In addition, centers brought value by “failing fast” and allowing institutions to rapidly shift resources to more effective areas. Deliberate capture of and recognition of these nonfinancial benefits underpinned many centers’ long-term sustainability. As center leaders noted, however, the nonquantifiable nature of these benefits meant that centers had to be deliberate in bringing them to the attention of leadership and making the case for their value. At our own institution, we have also been explicit about these benefits.^13,14^ Bindman and colleagues^15^ have noted that those seeking federal funding for innovation work face similar challenges.

### Limitations

Our study has some limitations. It was qualitative, designed to sample maximum variation, and was not representative of all innovation centers across the US. Care should thus be taken in generalizing conclusions. Due to COVID-19 limiting in-person case study visits, interviews were conducted over the telephone, often with only 1 representative from each center, limiting the variety of views gathered from each center. We spoke with center staff only, and not with institutional leaders, who might not share the same view of success or sustainability metrics. Last, interviews were (with one exception) conducted with centers that were still in existence (ie, had not failed). Although this is beneficial for exploring how successful innovation centers have managed to implement ideas, and provides some external evidence that center staff opinions of sustainability are valid, it may limit our understanding of the barriers to success or the major problems that inhibit long-term sustainability. To mitigate this limitation, we deliberately sought out examples of failed activities or projects in each interview with successful centers.

## Conclusions

In this qualitative study, we found that innovation centers can become sustainable entities within US health care institutions but that centers that survive over years have effectively worked with their institutions to define success more broadly than purely financial return on investment. It will be important to the long-term success of this paradigm for the field to develop more robust means of measuring these nonfinancial benefits. Moreover, we found that success is often dependent on maintaining a careful balance between working as insiders and working as outsiders, a design feature that should be explicitly considered when starting a new center.

## Notes

Supplement 1. **eAppendix 1.** Interview Guide **eAppendix 2.** CFIR Framework **eAppendix 3.** Innovation Centers Included in Study

Supplement 2.
Data Sharing Statement
