# Supplementary material for: Perceptions of Factors Associated With Sustainability of Health Care Innovation Centers
Source: JAMA Netw Open. 2023 Oct 27;6(10):e2339129. doi: 10.1001/jamanetworkopen.2023.39129 (PMC10611988; doi:10.1001/jamanetworkopen.2023.39129)
Supplement: Supplement 1. — eAppendix 1. Interview Guide eAppendix 2. CFIR Framework eAppendix 3. Innovation Centers Included in Study [file jamanetwopen-e2339129-s001.pdf]

Supplementary Online Content

Krelle H, Martinez M, Garry K, Horwitz LI. Perceptions of factors associated with sustainability of health care innovation centers. *JAMA Netw Open.* 2023;6(10):e2339129. doi:10.1001/jamanetworkopen.2023.39129

eAppendix 1. Interview Guide
eAppendix 2. CFIR Framework
eAppendix 3. Innovation Centers Included in Study

This supplementary material has been provided by the authors to give readers additional information about their work.

© 2023 Krelle H et al. *JAMA Network Open.*

eAppendix 1. Interview Guide

Interview topic guide and prompts

- Please outline the structure and funding of your innovation centre
  Prompts: size; types of staff; place within the wider university; funding sources

- What are the objectives of your innovation centre?
  - *What outcomes do you prioritise? Prompts: efficiency; quality; clinician experience; involvement of marginalized groups*
  - *What methods do you use? Prompts: QI, rapid RCTs, design-thinking*
  - *What financial/ economic outcomes do you have to demonstrate?*

- What have been the outcomes of your innovation centre?
  - *How do you define or measure success?*
  - *How does your institution define or measure success?*
  - *What is the right level/ quantity of failure?*
  - What is an example of a project that:
    - *Succeeded*
    - *Failed?*
- What have been the key ingredients to success at your centre?
  - *How is power distributed? What hard and soft levers do you use? How does this vary from innovation, to implementation, and evaluation?*
  - *What has worked well? What has worked less well? Prompts: the role of individuals; skills; structures; funding; external factors*
  - *What would you change?*
- What have your major challenges been?
  - *Funding; Timeline; Evaluation; Measurement; Collaboration with front-line; Implentation; Senior support; Prioritization*
- How has COVID19 affected how your center works?
  - *Did it change your objectives? Can you give an example of a project?*
  - *Did it change the way you worked? What was different? What worked better? Or less well?*
  - *Did it change the outcomes you were able to achieve?*

© 2023 Krelle H et al. *JAMA Network Open.*

eAppendix 2. CFIR Framework

Table 1: Analysis framework (adapted from CFIR)[4]

Overarching theme	Sub-theme
**Intervention characteristics:** what makes an implementable innovation?	
**Beyond the hospital:** wider community and influences	
**Outer setting:** what does the rest of the hospital need to look like to support the innovation centre?	Structures & networks (size/ social architecture/ age/ maturity/ history of collaboration etc)
	Culture (including culture of implementation/ innovation)
	Organisational incentives/ objectives/ priority put on innovation
	Financing
	ROI
	Leadership engagement
	Other
**Inner setting**: what are the success factors/ barriers within the innovation centre?	Team structure (including skill mix etc)
	Culture & ways of working (e.g. use of specific techniques/ lean/ design thinking)
	Location within/outside hospital structure
	Access to knowledge and info/ data
	Objectives
	Other
Characteristics of **individuals** involved in the centre	In the wider hospital
	In the centre itself
**Process**	Planning, engaging, selecting
	Implementing
	Evaluating
**Examples of success & failure**	Examples of success
	Examples of failure

eAppendix 3. Innovation Centers Included in Study

Brigham & Women's Clinician Innovation Group, Boston, MA [now Mass General Brigham Innovation]
Community Launchpad, Community Health Network, Indianapolis, IN
Evans Center for Implementation and Improvement Sciences, Boston University, Boston, MA
Geisinger Nudge Unit & The Steele Institute for Health Innovation, Danville, PA
Kaiser Permanente Garfield Innovation Center, San Leandro, CA
Ohio State University Medical Center IDEA Studio in Healthcare and Design, Columbus, OH
Pieces Technologies; partner with Parkland Health & Hospital System, Dallas, TX
USC Stevens Center for Innovation, Los Angeles, CA
Yale Center for Innovation Science, New Haven, CT
