# Supplementary material for: Perceptions of Factors Associated With Sustainability of Health Care Innovation Centers
Source: JAMA Netw Open. 2023 Oct 27;6(10):e2339129. doi: 10.1001/jamanetworkopen.2023.39129 (PMC10611988; doi:10.1001/jamanetworkopen.2023.39129)
Supplement: Supplement 2. — Data Sharing Statement [file jamanetwopen-e2339129-s002.pdf]

Data Sharing Statement

Krelle. Perceptions of Factors Associated With Sustainability of Health Care Innovation Centers. *JAMA Netw Open*. Published October 23, 2023. doi:10.1001/jamanetworkopen.2023.39129

Data
Data available: No
